# Antibacterial Regularity Mining Beneath the Systematic Activity Database of Lipopeptides Brevilaterins: An Instructive Activity Handbook for Its Food Application

**DOI:** 10.3390/foods11192991

**Published:** 2022-09-26

**Authors:** Yangliu Liu, Panpan Han, Yingmin Jia, Zhou Chen, Siting Li, Aijin Ma

**Affiliations:** Lab of Enzyme and Protein Engineering, School of Food and Health, Beijing Technology and Business University, No. 33 Fucheng Rd., Haidian District, Beijing 100048, China

**Keywords:** antibacterial activity, antibacterial peptide, *Brevibacillus laterosporus*, brevilaterins, food safety

## Abstract

Bacterial contamination is a primary threat to food safety. Therefore, the persistent development of natural antibacterial agents has become essential work. The present essay attempts to establish a systematic antibacterial activity database to instruct the food application of brevilaterins, promising antibacterial lipopeptides from *Brevibacillus laterosporus* S62-9. Minimum inhibitory concentrations (MIC) and minimum bactericidal concentrations (MBC) were systematically collected from 43 species of standard bacteria and 140 strains of isolated bacteria (food spoilage bacteria and antibiotic-resistant bacteria) using a broth dilution method. The results showed that brevilaterins performed a broad-spectrum inhibitory (0.5~128 μg/mL) and bactericidal activity (1~256 μg/mL), especially efficient against Gram-positive bacteria and spoilage bacteria from grain products. Moreover, brevilaterins not only inhibit and kill multiple antibiotic-resistant bacteria but do not readily develop resistance, with a small specific value of MBC/MIC (1~8). Furthermore, brevilaterins would interact with negatively charged sodium dodecyl sulfate and bind amphipathic soybean phospholipid with an affinity constant of *K_D_* = 4.70 × 10^−4^ M. No significant activity difference was found between brevilaterin B and brevilaterin C. Collectively, this work contributed rich antibacterial data of brevilaterins and revealed the antibacterial regularity beneath these data, which can be used as an activity handbook to instruct their application in food safety.

## 1. Introduction

Microbial contamination, especially bacteria, has been a most serious threat to food safety. On a global scale, 90% of cases of food poisoning are caused by foodborne pathogenic bacteria, such as *Campylobacter*, *Listeria monocytogenes*, *Staphylococcus aureus*, *Bacillus cereus*, and *Salmonella* [1,2]. In general, species of food-borne bacteria varied in different food systems or food processing from farm to table. For example, *Bacillus* has a proteolytic ability; thus, widely polluting protein-rich foods, such as meat, fish, eggs, and dairy products [3,4]. *Listeria* and *Pseudomonas* are the primary pathogenic bacteria existing in chilled foods and ready-to-eat food that cause acute gastroenteritis [5,6]. Moreover, new emerging drug-resistant bacteria may spread via the food chain, which also increases the risk to food safety and public health [7,8]. Therefore, it is important to develop suitable efficient preservatives to combat specific spoilage bacteria, food-borne bacteria, and even drug-resistant bacteria in different food systems.

Recently, natural antimicrobial peptides from *Brevibacillus* have gained extensive attention again since new lipopeptides, brevicidines, have been discovered to be efficient, particularly against Gram-negative bacteria, through global genome mining technology [9]. These *Brevibacillus* peptides are generally grouped with a similar primary structure, such as bogorol A~L [10,11], brevibacillin I/V/2V [12,13], brevicidin A/B, relacidine A/B [14,15], and brevilaterin A~E as well V1~V6 [16,17]. Among them, brevilaterins are a group of antimicrobial lipopeptides discovered in our previous research. They have been demonstrated to be synthesized by a non-ribosomal peptide synthetase system in *Brevibacillus laterosporus* S62-9 with the primary structure as Hmp-Aba-V/M-Orn-V/I-V-V/I-K-V-L-K-Y-L-Volinol [18]. Their outstanding antibacterial activity, as well as low hemolysis, make them promising for development in the food industry and agriculture.

Before the commercial food application of an antibacterial peptide, more basic information about antibacterial activity should be provided. As we know, the concept of antibacterial activity contains inhibitory and bactericidal effects. Nevertheless, most of the current research is just concerned with inhibitory activity, such as minimal inhibitory concentration (MIC) [19,20,21,22]. We know little about bactericidal effects and the relationship between inhibitory and bactericidal activity. In addition, as pathogenic bacteria varied in different food systems, activity investigations on sufficient species of food-borne pathogenic bacteria would provide more comprehensive instruction for food application. Yet, only a small minority of standard bacterial species (less than 15) were investigated as indicators of these *Brevibacillus* antimicrobial peptides, such as the most classic *Escherichia coli*, *S. aureus, B. subtilis*, and *L. monocytogenes* [2,9,23]. The available results seemed unable to reflect the overall perspective of antibacterial activity to guide the actual application of a *Brevibacillus* antibacterial peptide.

Herein, to lay a sound theoretical foundation for the food application, this research systemically studied the antibacterial regularity of brevilaterins. We collected the data information on MIC and minimum bactericidal concentration (MBC) of brevilaterins against a variety of bacteria to reveal the antibacterial regularity of brevilaterins beneath the database. Indicators included 50 strains of standard bacteria, 70 strains of bacteria isolated from common spoilage food, and 70 isolated strains of drug-resistant bacteria, respectively. In the present study, which aim to analyze the relationship between antibacterial activity and structure, brevilaterins refer to brevilaterin B and brevilaterin C. The interaction of brevilaterins with food compositions, negative-charged sodium dodecyl sulfonate (SDS) (a food additive, 21 CFR 172.822) [24], and amphipathic soybean phospholipid, are further explored. These analysis results will present a more sound and valuable antibacterial regularity handbook for brevilaterins, in order to better instruct their food application.

## 2. Materials and Methods

### 2.1. Brevilaterins Stock Solution

Brevilaterins from *Br. laterosporus* S62-9 were extracted and purified as described in our previous research [17]. The primary structure of brevilaterin B and brevilaterin C are determined as Hmp-Aba-M-Orn-I-V-V-K-V-L-K-Y-L-Valinol and Hmp-Aba-V-Orn-I-V-V-K-V-L-K-Y-L-Valinol by NMR, respectively. Brevilaterin B (98.83% of purity) and brevilaterin C (93.16% of purity) were dissolved in a phosphate buffer solution (PBS, 10 mM, pH 7.4) to prepare 10 mg/mL of stock solutions and kept at −20 °C until use.

### 2.2. Indicator Bacteria and Medium

#### 2.2.1. Standard Bacteria

A total of 50 strains of standard bacteria were selected as indicators. These bacteria belong to 43 species and 25 genera, which covered the majority of species of representative food-borne pathogens and spoilage bacteria reported in different common foods. Meanwhile, these bacteria differ in morphology, referring to Gram-positive and Gram-negative, cocci, bacilli, and whether they are spore producers, which can be used for the multi-angle analysis of the antibacterial difference. All of the bacteria were purchased from the China Center of Industrial Culture Collection (CICC) (Beijing, China) and cultured according to CICC instructions, as shown in Table S1.

#### 2.2.2. Isolated Bacteria from Spoilage Food

To further verify the antibacterial regularity of brevilaterins, 70 strains of dominant bacteria were initially isolated from 29 kinds of natural perishable foods using a nutrient broth medium at 37 °C (Table S2). These foods were purchased from a supermarket and mainly classified into four types according to their main ingredients, including animal foods (meat, eggs, and dairy products), soybean products (bean curd, fermented bean curd, and soybean milk), grain products (fresh noodles, bread, bun, rice, and purple potato), and fruits and vegetables (mango, apple, pitaya, Chinese cabbage, lettuce, and pickle). Bacteria isolated from these spoilage foods were preliminarily classified by the Gram-staining method and kept in our lab for the test.

#### 2.2.3. Isolated Bacteria to Resist Antibiotics

A total of 70 strains of drug-resistant bacteria were used as indicators to analyze the activity of brevilaterins against drug-resistant bacteria. These bacteria were screened by a nutrient broth medium at 37 °C to resist one antibiotic or compound antibiotics, including methicillin, penicillin, vancomycin, teicoplanin, meropenem, and ciprofloxacin (Table S3).

### 2.3. MIC and MBC Determination and Quality-Control Test

MICs were determined using the broth microdilution assay with some modification [25]. Briefly, 1024 μg/mL of brevilaterins solutions were diluted using the medium in 96-well microtiter plates by a two-fold dilution series. The final volume of brevilaterins solutions in each well was 100 µL (final concentration range of 0.5~256 μg/mL). Then, each well was inoculated with 100 µL of the indicator bacteria suspension (final concentration of 5 × 10^5^ CFU/mL in well), adjusting by the *Maxwell* 0.5 standard turbidimetric tube method in 0.85% of normal saline solution. The MIC value was defined as the lowest concentration of brevilaterin with no visible turbidity after 16~24 h of incubation at the optimal temperature (37 °C for isolated strains). Thereafter, 100 μL of culture at a concentration higher than MIC were paved onto an agar medium (nutrition agar for the isolated strains) and incubated for 24 h at the optimal temperature (37 °C for isolated strains). The MBC was defined as the lowest concentration at which viable bacteria are less than five. The experiments were carried out in triplicate.

In order to ensure the reliability of the MIC measurement system, quality-control strains and agents were taken as the reference according to CLSI-M07 A9 [26]. Herein, daptomycin or vancomycin against both *S. aureus* subsp. *aureus* ATCC 29213 and *Enterococcus faecalis* ATCC 29212 were taken as controls for Gram-positive bacteria. Polymyxin against *P. aeruginosa* ATCC 27853 and *E. coli* ATCC 25922 was used as control for the Gram-negative inhibition system in each batch of experiments. When these MICs are within the scope of quality control range, this experiment system is considered feasible.

### 2.4. Relative Antibacterial Activity Affected by Food Compositions

The antibacterial activity of brevilaterin B in SDS (BN35750, Biorigin, Beijing, China) solution or soybean phospholipids (Macklin Co. Ltd., Shanghai, China) was determined using the agar diffusion method to explore the interaction [27]. In brief, 0.6 mg/mL of brevilaterin B solution was mixed with the same volume of SDS aqueous solution (0.01~0.1% (m/V)) or soybean phospholipids solution (0.2~2% (m/V)). Then, 50 μL of the mixture was added into an Oxford cup on a pre-cooled agar plate containing *S. aureus* ATCC 25923 suspensions (~10^7^ CFU/mL). After incubation for 24 h at 37 °C, the inhibition zone was recorded and the antibacterial activity of brevilaterin B without treatment was regarded as 100%. The experiments were carried out three times and shown as the mean ± standard deviation values.

### 2.5. Surface Hydrophobicity

The surface hydrophobicity assay was carried out using a 8-anilino-1-naphtalene sulfonic acid (ANS) probe method [28]. Briefly, 0.6 mg/L of brevilaterin B was initially mixed with SDS solution to 4 mL of total volume. Then, 20 μL of 8 mM of ANS probe (Sigma-Aldrich, St. Louis, MO, USA) solution was added into the mixture. After dark incubation for 10 min at 25 °C, the fluorescence of each sample was measured on a Hitachi F-7100 fluorescence spectrometer (Hitachi, Tokyo, Japan) three times, with 370 nm of the excitation wavelength and 5 nm of slit width at a scanning speed of 1200 nm/min. The surface hydrophobicity index (*S*_0_) was defined as the slope of the fitted curve, where the relative fluorescence intensity *F* at 480 nm responded to the concentration of brevilaterin solution. The experiments were repeated in triplicate and shown as the mean ± standard deviation values.

### 2.6. Circular Dichroism Spectrum

The circular dichroism spectrum of brevilaterin B was observed on a Bio-logic Mos-500 spectropolarimeter (Claix, France). Briefly, 0.6 mg/L of brevilaterin B in SDS solution or soybean phospholipids was scanned from 190 nm to 250 nm in a 0.1 cm path-length cell. The scan speed was 50 nm/min, and the bandwidth was 1 nm. Each spectrum was obtained by subtracting the corresponding solvent blank and averaged three scans. The percentage of secondary conformations was finally predicted using SELFCON II software in the equipment. The experiments were repeated in triplicate.

### 2.7. Surface Plasmon Resonance

The binding interaction between brevilaterin B and soybean phospholipids was further demonstrated on four-channel surface plasmon resonance (4SPR, Reichert Technologies, Depew, NY, USA) equipment. At first, EDC/NHS solution (40 mg/10 mg dissolving in 1 mL) was run through channel 3 and 4 at a speed of 10 μL/min for 7 min. Then, 30 μg/mL of brevilaterin in 10 mM of NaOAc solution (pH 4.5) was captured on the planar Mixed SAM surface (13206061, Reichert Technologies, Depew, NY, U.S.A.) by running at 10 μL/min for 8 min. After that, 1 M of ethanolamine solution (pH 8.5) was run through channel 3 and 4 for 4 min to clean the system. For the mobile phase, soybean phospholipids solutions (600 μM to 3.75 μM) were prepared with 1% PBST solution (containing 1% ethanol) in a two-fold serial dilution. The diluted phospholipids solution was injected at a speed of 25 μL/min in concentration from low to high taking 1% PBST solution as a blank. The whole binding process lasted for 1 min and the dissociation process was performed for 2 min. Finally, the obtained data were analyzed by TraceDrawer software 1.9.2 to calculate the affinity constant *K_D_* using a 1:1 binding model. The experiments were carried out in triplicate.

## 3. Results and Discussion

### 3.1. Quality Control of MIC Determination System

To ensure the reliability of the MIC determination system, a quality control experiment was carried out with daptomycin against two Gram-positive bacteria and polymyxin sulfate B against two Gram-negative bacteria according to CLSI-M07 A9 [26]. In Table 1, both MICs of daptomycin against *S. aureus* subsp. *aureus* ATCC 29213 and *E. faecalis* ATCC 29212 were in their respective quality range. Likewise, the experimental MIC values of polymyxin sulfate B against *P. aeruginosa* ATCC 27853 and *E. coli* ATCC 25922 met the quality requirements. Therefore, the results indicated our MIC experimental system was reliable and feasible in follow-up trials.

### 3.2. Antibacterial Regularity against Standard Bacteria

Extensive coverage of bacterial species will enable us to comprehensively understand the antibacterial regularity of brevilaterins. Herein, 50 strains of standard bacteria from 43 species and 25 genera were used as indicators to collect MIC and MBC values, as shown in Table 2, which covered most of the food spoilage and food-borne pathogenic bacteria in the food industry. Both the inhibitory and bactericidal regularity of brevilaterins were further analyzed in the following Section 3.2.1 and Section 3.2.2 based on these data.

#### 3.2.1. Inhibitory Property of Brevilaterins

The MIC value is a vital parameter to evaluate the inhibitory activity of new antibacterial agents and determine a strain’s susceptibility to an antibacterial agent [29]. Overall, brevilaterin B and brevilaterin C showed general inhibition activity against most of the tested bacteria, even MRSA and VRE, with an MIC range of 0.5~128 µg/mL (Table 2 and Table 3). When the bacteria were classified as Gram-positive (26 strains) and Gram-negative bacteria (24 strains), more activity properties of brevilaterins were revealed further.

**Figure 1:**
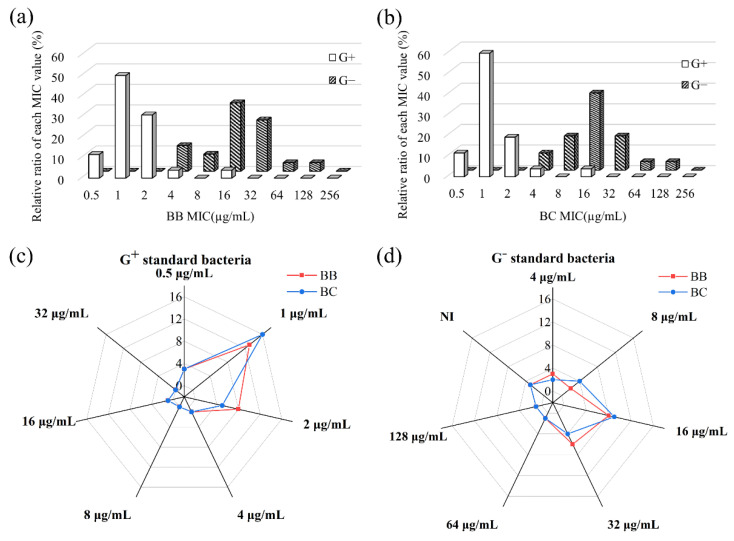
Inhibitory activity of brevilaterins against 50 species of standard bacteria. (**a**,**b**) the distribution of minimal inhibitory concentration (MIC) values of brevilaterin B (BB) and brevilaterin C (BC) against all standard bacteria; (**c**,**d**) comparison of MIC value distribution of brevilaterin B and brevilaterin C against Gram-positive bacteria (**c**) and Gram-negative bacteria (**d**), respectively. G^+^ stands for Gram-positive. G^−^ stands for Gram-negative, for convenience.

Brevilaterins can inhibit all tested Gram-positive bacteria with MIC values in the range of 0.5~16 μg/mL (Figure 1a,b). Over half of the tested Gram-positive bacteria were inhibited under 1 μg/mL, and 90% of them were susceptible at 2 μg/mL (Table 3), while these two values were 16 μg/mL and more than 128 μg/mL for G^-^ bacteria, respectively. Almost a 16-fold significant difference between Gram-positive and Gram-negative bacteria reflected the efficient antibacterial activity of brevilaterins to Gram-positive bacteria. The effects of brevilaterins on Gram-positive bacteria are similar to those of commercial peptide antibiotics (vancomycin and daptomycin) (Table 1), suggesting excellent inhibitory activity and commercial development potential.

Furthermore, for specific bacterial genera and species, brevilaterins performed efficient inhibitory activity on *Bacillus*, *Staphylococcus*, *Listeria*, *Enterococcus*, *Micrococcus*, *Streptococcus*, *Lactococcus*, *Leuconostoc*, and *Lactobacillus* at MICs of 0.5~2 µg/mL. Among them, *Micrococcus luteus* CICC 10396, *Bacillus coagulans* CICC 20138, and *Bacillus megaterium* CICC 10448 were the most susceptible to brevilaterins with MIC of just 0.5 µg/mL (Table 2).

In the food industry, pathogenic or spoilage *Bacillus* is a tough problem for meat, soybean, and grain products, because they can produce spores to survive in a tough environment and have a strong ability to break down protein and fat causing spoilage once the conditions are qualified [30,31]. *Bacillus cereus*, for example, can produce enterotoxin resulting in food poisoning, especially common in overnight rice [32]. Thus, *Bacillus* is an important representative genus in order to evaluate antibacterial property. In this study, brevilaterins have a significant bacteriostatic activity to all *Bacillus* spp. (*B. coagulans*, *B. megaterium*, *B. cereus*, *B. pumilus*, *B. fusiformis*, *B. subtilis*, and *B. subtilis* subsp. *Subtilis*) at MIC of 0.5~2 µg/mL (Table 2). The MIC value against *B. cereus* was similar to brevibacillin family peptides (Bre, Bre V, Bre I, and Bre 2V) and bogorols-family peptides (bogorol K and bogorol L) at an MIC of 1~2 µg/mL [10,17], and it was lower than the MIC of commercial preservative nisin or poly-L-lysine against *B. subtilis* [33]. These findings suggested the advantage of brevilaterins to control *Bacillus* spp. in protein-rich foods as well as grain products.

Furthermore, for Gram-positive bacteria, no significant inhibition difference between bacillus (12 strains) and coccus bacteria (14 strains) was found. Both bacillus and coccus bacteria showed intensive MIC values at 0.5~2 µg/mL (Table 2), which indicated the activity of brevilaterins was not affected by the bacterial shape. Only *Paenibacillus polymyxa* CICC20128 was the least susceptible to brevilaterins with an MIC of 16 µg/mL, which was different from other Gram-positive bacteria. A possible explanation is that *P. polymyxa* may have a natural tolerance to the inhibitory action of brevilaterins, because it itself may produce antibacterial agents [34]. Finally, it is noteworthy that brevilaterin also exhibited an efficient inhibitory activity to MRSA (MIC of 4 µg/mL) and VRE (MIC of 1 µg/mL), implying its alternative potential in inhibiting resistant bacteria.

For Gram-negative bacteria, brevilaterin showed moderate bacteriostatic activity with MICs of 4~128 µg/mL as compared with other reported antibacterial peptides from *Brevilbacillus* [14,15]. Among them, *Acinetobacter baumannii* CICC 10980, *Alcaligenes faecalis* CICC 10981, *Shewanella putrefaciens* CICC 22940, *Shigella dysenteriae* CICC 23839, and *E. coli* ATCC 25922 were the most susceptible bacteria with an MIC of 4~8 µg/mL, which were close to the MIC values of Gram-positive bacteria (Table 2). Meanwhile, more than half of the Gram-negative bacteria could be inhibited at an MIC of 16~32 µg/mL, as shown in Figure 1, including *Pseudomonas*, *Shewanella*, *Escherichia*, *Klebsiella*, *Cronobacter*, *Virbrio*, *Citrobacter*, *Yersinia*, and *Salmonella* (Table 1). In actual food, *Virbrio*, *Shewanella*, and *Salmonella* are common pathogens in aquatic products [35,36]. *Yersinia*, especially *Y. enterocolitica*, is a unique psychrophilic pathogenic bacterium, often appearing in animal foods, especially dairy products transported or stored at 0~5 °C [37,38]. Although the inhibitory activity of brevilaterins was far more for some antibacterial agents specific to Gram-negative bacteria, such as relacidines, brevicidines, and polymyxin B, the results showed that brevilaterins have a better inhibitory activity on Gram-negative bacteria than nisin [14,15]. Unfortunately, we did not obtain the MICs to *Serratia marcescens* CICC 10898, *Proteus mirabilis* CICC 21516, and *Proteus vulgaris* CICC 10866, even at the maximum concentration of 256 µg/mL. The reason for this is not clear, but it was speculated that *Serratia* and *Proteus* may be naturally resistant to peptide antimicrobial agents, as discovered in some clinical trials [39]. This result, combined with the action on *P. polymyxa* CICC20128, may also provide an interesting revision strategy to study the antibacterial mechanism of brevilaterins.

As demonstrated in the radar map, Figure 1c,d, the activity difference of brevilaterin B and brevilaterin C were compared. The MICs of these two peptides against Gram-positive bacteria (Figure 1c) or Gram-negative bacteria (Figure 1d) were broadly overlapped, showing no significant activity differences between different components. Structurally, brevilaterin B and brevilaterin C just differ at the second amino acid residue. Therefore, the similarity in antibacterial activity suggested that the change at the second amino acid would not significantly affect their same activity center. Other antibacterial peptides from one family are also reported to have similar activity against the same bacteria [40,41]. This subtle structural change mechanism in some ways may prevent resistance to improve the overall defense capabilities of peptide groups [11].

#### 3.2.2. Bactericidal Property of Brevilaterins

MBC is a vital parameter to evaluate the bactericidal capacity of antibacterial agents. On the whole, brevilaterins showed universal bactericidal activity to almost all test spoilage and pathogenic bacteria with the MBC range of 0.5~256 μg/mL, except for the three Gram-negative bacteria with undetectable MICs and MBCs (Table 2 and Table 3).

**Figure 2:**
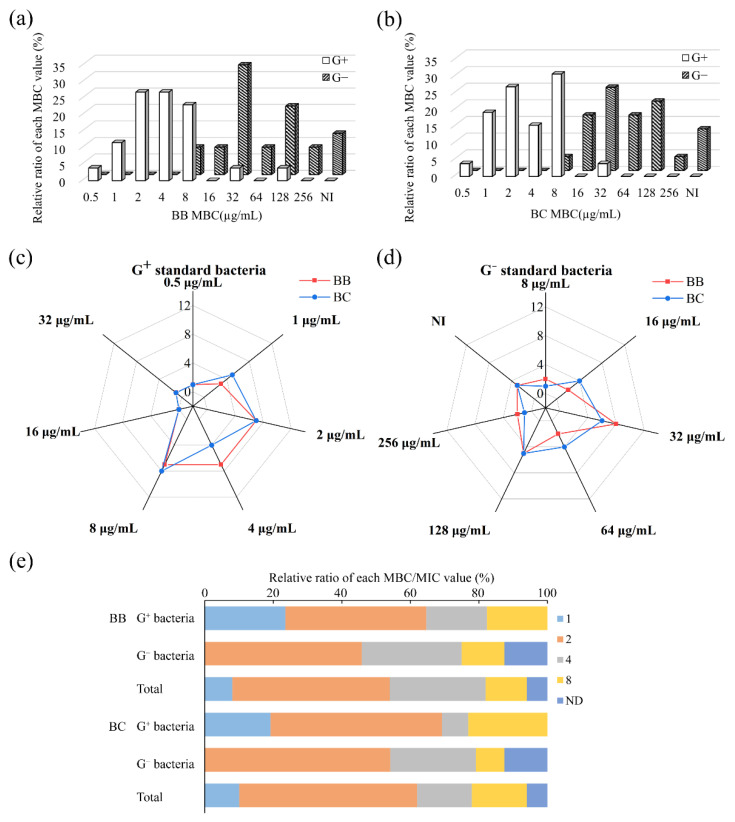
Bactericidal activity of brevilaterins against 50 species of standard bacteria. (**a**,**b**) the distribution of minimal bactericidal concentration (MBC) values of brevilaterin B (BB) and brevilaterin C (BC) against all standard bacteria; (**c**,**d**) comparison of MBC value distribution of brevilaterin B and brevilaterin C against Gram-positive bacteria (**c**) and Gram-negative bacteria (**d**), respectively; (**e**) the relative ratio of MBC/MIC value of brevilaterins against bacteria. ND, no value for undetected MIC and MBC. G^+^ stands for Gram-positive. G^−^ stands for Gram-negative, for convenience.

Similar to bacteriostatic properties, brevilaterins exhibited a more efficient bactericidal ability against Gram-positive bacteria than Gram-negative bacteria (Figure 2a,b). Almost half of Gram-positive bacteria were killed at 2 μg/mL (brevilaterin B) or 4 μg/mL (brevilaterin C), and 90% of them were lethal at 8 μg/mL. Nevertheless, half of Gram-negative bacteria were killed at 32 μg/mL (brevilaterin B) or 64 μg/mL (brevilaterin C), respectively (Table 3). The 16-fold difference between Gram-positive bacteria and Gram-negative bacteria, similarly to MICs, showed the efficient bactericidal activity on Gram-positive bacteria, but no significant activity difference was found between brevilaterin B and brevilaterin C (Figure 2c,d).

Furthermore, the specific value of MBC/MIC was analyzed to reflect the relationship between inhibitory and bactericidal activity. When the value is under 4, this indicates a good bactericidal activity [42]. In Table 2, brevilaterins exhibited the most efficient antibacterial activity against *B*. *megaterium*, *B*. *subtilis* subsp. *subtilis* ATCC 6051, and *S. aureus* subsp. *aureus* ATCC 6538 with the same value of MIC and MBC. This phenomenon was only found in Gram-positive bacteria, indicating that Gram-positive bacteria were more susceptible to brevilaterins. On the other hand, “selective pressure” caused by concentration is regarded as the main factor to develop drug-resistance; thus, the value of MBC/MIC may also be used to evaluate the possibility to developing drug resistance. When this value is up to 32, one bacterium may possess or be under feasible threat of developing resistance to this antibacterial agent [43,44]. Thus, under this condition, the antibacterial agent was risky and should be considered twice in this application. In this research, the most specific values of MBC/MIC were no more than 8. Over 60% of Gram-positive bacteria and over 45% of Gram-negative bacteria would be killed with under 2 of MBC/MIC (Figure 2e), which is quite a small value for bacteria to develop drug-resistance to brevilaterins.

### 3.3. Verification of Antibacterial Regularity on Isolated Bacteria

Based on the analysis in Section 3.2, we concluded several important antibacterial regularities of brevilaterins. To further verify the antibacterial regularities, 140 strains of isolated bacteria, including 70 strains from different spoilage food and 70 strains resistant to antibiotics, were used as indicator bacteria here.

#### 3.3.1. Antibacterial Property against Food Spoilage Bacteria

**Figure 3:**
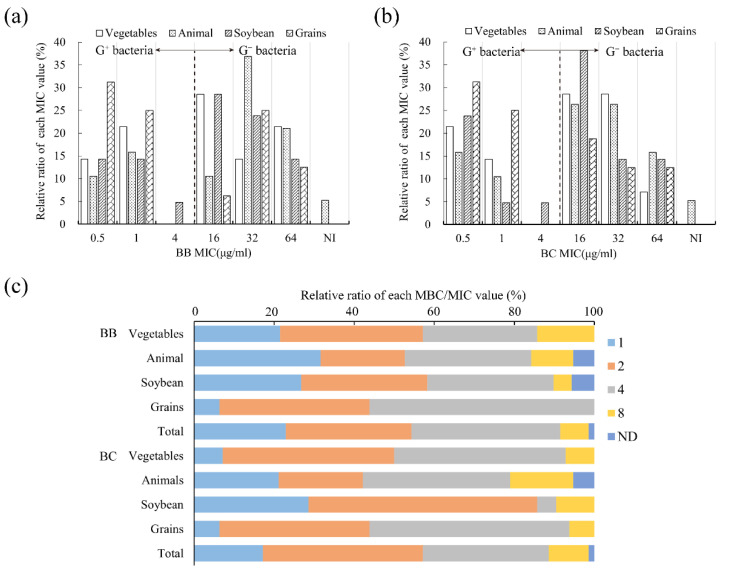
Antibacterial activity of brevilaterins against 70 strains of isolated spoilage bacteria. (**a**,**b**) the distribution of MIC values of brevilaterin B (BB) and brevilaterin C (BC) against all isolated spoilage bacteria. G^+^ and G^−^ stand for Gram-positive and Gram-negative, respectively, for convenience; (**c**) the relative ratio of MBC/MIC value of brevilaterins against spoilage bacteria from different foods. ND, no value for undetected MIC and MBC.

Different types of foods would breed different spoilage bacteria. Herein, we determined MIC and MBC values against 70 strains of dominant isolated bacteria from four types of spoilage foods, including animal foods, soybean products, grain products, and vegetables and fruits (Table S2). Overall, the MIC and MBC values ranged between 0.5~64 μg/mL and 1~256 μg/mL, respectively. Only one Gram-negative bacteria Q2-3-1 detected no activity (Table 4). It proved brevilaterins have widespread inhibitory and bactericidal action on spoilage bacteria from common foods. Similar to standard bacteria, both brevilaterin B and brevilaterin C were obviously efficient against Gram-positive bacteria with MICs concentrated at 0.5~1 μg/mL, while that of Gram-negative bacteria was 16~64 μg/mL (Figure 3a,b). In addition, 90% of the specific ratio of MBC/MIC were distributed at 1, 2, and 4 (Figure 3c). The small specific value not only suggested brevilaterins have good bactericidal ability but were also less able to develop drug resistance.

According to the proportion of each MIC value, brevilaterins were more efficient against spoilage bacteria from grain products with an MIC_50_ of 1 μg/mL. Then, susceptibility of bacteria from soybean products, and vegetables and fruits were secondary with an MIC_50_ of 16 μg/mL, and spoilage bacteria from animal foods were less susceptible to brevilaterins with an MIC_50_ of 32 μg/mL (Figure 3a,b). In general, bacteria can selectivity break down food ingredients. Grain products rich in carbohydrates, such as rice and flour-based products, are feasibly able to be polluted by *B. subtilis*, *B. cereus*, *Lactobacillus*, *S. aureus*, and *E. coli* [45]. These bacterial species were susceptible to brevilaterins with an MIC of 1~16 μg/mL, as revealed in Section 3.2. Vegetables and fruits rich in carbohydrates, such as fiber, usually have a short storage life after harvest, even at 0~4 °C. The common contaminant bacteria are *L. monocytogenes*, *Pseudomonas*, *Leuconosto*, pathogenic *E. coli*, *Shigella* spp., and *Shewanella* spp. [46,47]. Meanwhile, animal foods (meat, eggs, and dairy products) and soybean products are rich in proteins and fat, becoming a natural medium for various bacteria. The primary contaminant bacteria are *Bacillus* spp., *S. aureus*, *Lactococcus*, pathogenic *E. coli*, *V. parahaemolyticus*, *Shigella dysenteriae*, *S. typhimurium*, and *Y. enterocolitica* [48,49]. Among them, Gram-negative bacteria are in a dominant position in rotten animal foods, but brevilaterins were moderately efficient against Gram-negative bacteria, as concluded in Section 3.2. Thus, the overall antibacterial activity of brevilaterins against bacteria from animal foods was not very encouraging. The less efficient activity in animal foods may be improved by concocting a combination with other antibacterial agents. For example, the combination of the use of brevilaterins and citric acid showed a synergistic effect on *E. coli* [50].

In food safety, choosing the appropriate antibacterial agents according to the specific spoilage bacteria in different foods is an effective strategy to achieve accurate control [51]. The above results have revealed that brevilaterins are promising for controlling bacterial contaminants from grain products, vegetables, fruits, and soybean products, but are less efficient for spoilage bacteria from animal foods. When combining the specific bacterial species, these findings can be used as an activity handbook to instruct the accurate application of brevilaterins in the food industry.

#### 3.3.2. Antibacterial Property against Antibiotic-Resistant Bacteria

**Figure 4:**
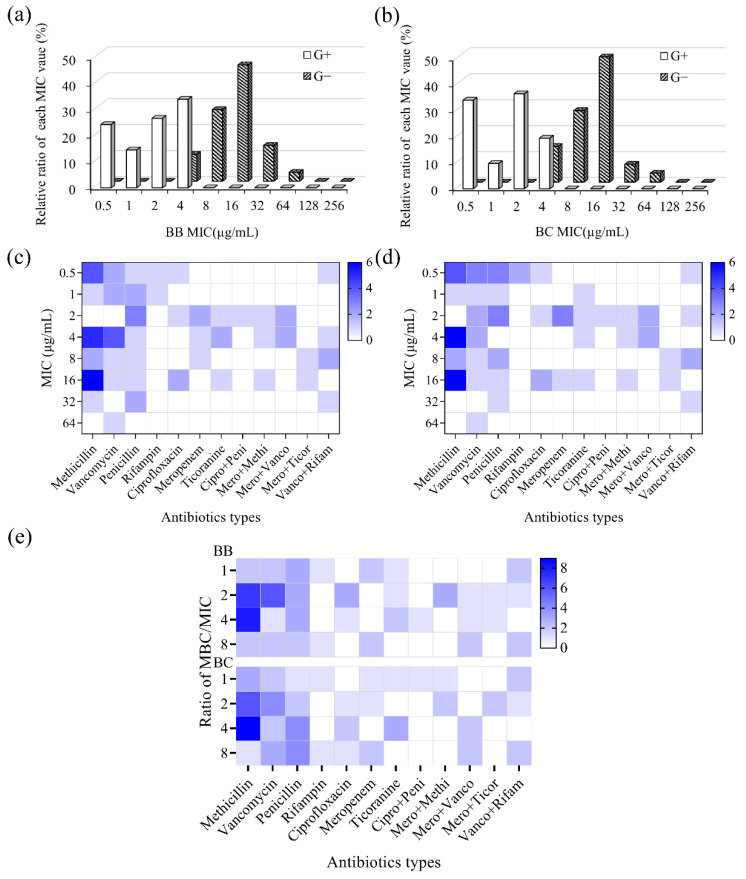
Antibacterial activity of brevilaterins against 70 strains of isolated antibiotic-resistant bacteria. (**a**,**b**) the distribution of MIC values of brevilaterin B (BB) and brevilaterin C (BC) against all bacteria. G^+^ and G^−^ stand for Gram-positive and Gram-negative, respectively, for convenience; (**c**,**d**) the distribution of MIC values of brevilaterin B (**c**) and brevilaterin C (**d**) against bacteria resistant to different antibiotics; (**e**) the relative ratio of MBC/MIC values of brevilaterins against isolated bacteria resistant to different antibiotics.

In previous results, we have found brevilaterins showed a good inhibitory action on MRSA and VRE. To verify and analyze the antibacterial property against antibiotic-resistant bacteria, MIC and MBC values against 70 strains of bacteria, resistant to different antibiotics (ciprofloxacin, methicillin, rifampicin, meropenem, penicillin, teicoplanin, and vancomycin), were recorded in Table S3. Overall, brevilaterins have a general inhibitory and bactericidal activity on various antibiotic-resistant bacteria with MICs in the range of 0.5~64 μg/mL and MBCs in the range of 0.5~128 μg/mL, respectively. Both brevilaterin B and brevilaterin C showed more efficient inhibitory activity to antibiotic-resistant Gram-positive bacteria (MIC of 0.5~4 μg/mL) than Gram-negative bacteria (MIC of 4~64 μg/mL), as shown in Figure 4a,b. In addition, brevilaterin B and C performed similarly on drug-resistant bacteria, which was in line with the findings for standard bacteria.

As previously known, the emerging antibiotic-resistant bacteria in the environment have become a problem worldwide. Notably, these antibiotic-resistant bacteria can also be transferred into food and the human body via the food chain, bringing a severe safety challenge for food safety [52]. Therefore, antibacterial peptides that combat antibiotic-resistant bacteria and drug resistance get much interest. In this study, brevilaterins were proved to have a general antibacterial activity to different antibiotic-resistant bacteria (Figure 4c,d), especially Gram-positive bacteria. Moreover, the specific MBC/MIC values of each bacterium was under 8 (Figure 4e), implying that brevilaterins were adversaries to the development of resistant bacteria. These findings were just what we expected: an antibacterial peptide active to antibiotic-resistant bacteria and with little possibility of developing resistance will decrease the potential safety risk. Thus, brevilaterins with these advantages are promising to face the challenge of antibiotic-resistant bacteria.

Furthermore, these antibacterial activity data could provide indirect evidence for the antibacterial mechanism, as these bacteria usually have evolved resistant genes to different antibiotics. Penicillin, methicillin, and meropenem are broad-spectrum β-lactam antibiotics to combat bacteria [53]. Vancomycin and teicoplanin are narrow-spectrum glycopeptide antibiotics against MRSA, *Listeria* spp., *Enterococcus* spp., and other Gram-positive bacteria by inhibiting the cell wall synthesis [54]. Rifampicin and ciprofloxacin are efficient bactericides to interfere with the biosynthesis of RNA and DNA [55,56]. The activity of brevilaterins against these isolated bacteria imply that the current action targets in the cell wall, RNA, or DNA may not be suitable for brevilaterins, which provided indirect evidence for the membrane mechanism [27].

### 3.4. Effect of Food Compositions on Brevilaterins

In the practical food system, antibacterial agents will not only combat specific bacteria, but may be affected by complex food compositions [57]. Considering positive charge and hydrophobicity are generally important factors for the membrane mechanism of brevilaterins [58], herein, we used negatively charged SDS and amphipathic soybean phospholipids as representatives to analyze the effect.

#### 3.4.1. Interaction with SDS

**Figure 5:**
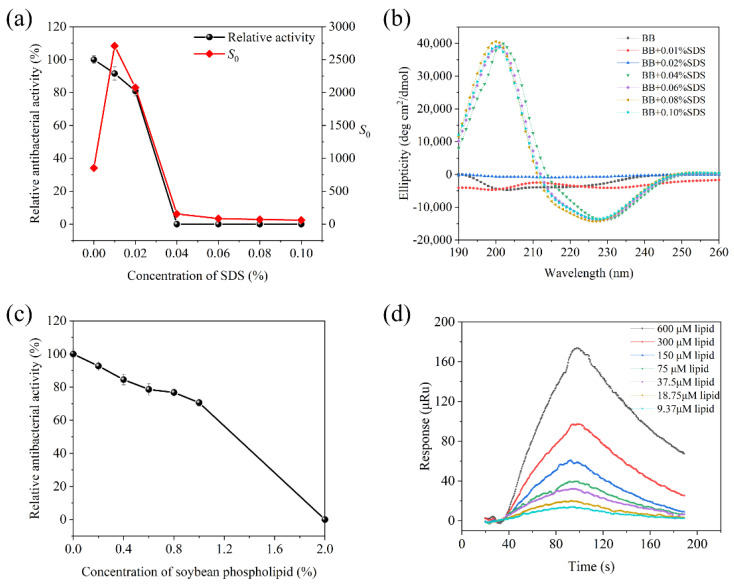
Effect of sodium dodecyl sulfate and soybean phospholipid on brevilaterin. (**a**) change of antibacterial activity and hydrophobicity *S*_0_ affected by sodium dodecyl sulfate. Values are the mean ± SD from three trials; (**b**) circular dichroism spectrum changes of brevilaterin B in sodium dodecyl sulfate solution; (**c**) antimicrobial activity change of brevilaterin B in soybean phospholipid solution. Values are the mean ± SD from three trials; (**d**) binding interaction curve between brevilaterin B and soybean phospholipid determined by surface plasmon resonance technology.

As the concentration of SDS solution increased, the antibacterial activity significantly decreased in Figure 5a. When the concentration was over 0.04%, the antibacterial activity was almost undetectable, suggesting that SDS have an inhibitory effect on brevilaterin. At the same time, the hydrophobic index *S*_0_ was observed with a similar decreased tendency, which indicated a good correlation between antibacterial activity and hydrophobicity. The changes in hydrophobicity were assumed to be related with the structure change. Thus, the secondary structure of brevilaterin was further studied by the circular dichroism spectrum. As shown in Figure 5b, brevilaterin mainly contained a random coil (53%) in PBS solutions, and the spectrum had no significant change in 0.01~0.02% of SDS solution. Nevertheless, at the inflexion point of 0.04%, brevilaterin has a significant positive peak at 202 nm and a negative peak at 225 nm, which demonstrated that brevilaterin changed its structure with the increased content of *β*-fold.

SDS is an anionic surfactant with one net charge, while brevilaterin B has three net positive charges (one ornithine and two lysine residues). Therefore, when the molar ratio of SDS to brevilaterin is up to 3:1 (that is 0.032% SDS), the charges of brevilaterin can be completely neutralized with SDS. In 0.01~0.02% of SDS solution, brevilaterin is in relative excess. Thus, the electrostatic interaction of brevilaterin and SDS molecular resulted in decreased antibacterial activity. At 0.04% of SDS solution, brevilaterin has been completely neutralized with undetected activity. Excess SDS molecular would form vesicle-like structures with hydrophobic fatty acid chains in the internal, which created a hydrophobic environment similar to bacterial cytomembrane [59]. In this environment, brevilaterin was supposed to be adsorbed on the surface and change its secondary structure from a random coil to a *β*-sheet.

#### 3.4.2. Interaction with Soybean Phospholipid

Soybean phospholipid is a mixture with a main component of phosphatidyl choline, also a non-ionic surface-active agent with hydrophilic and lipophilic properties [60]. Herein, it was used to certify the role of hydrophobicity interaction between brevilaterin and mimic a membrane environment. In Figure 5c, the antibacterial activity of brevilaterin significantly decreased as the concentration of soybean phospholipid increased to 2%. It seems that the soybean phospholipid may inhibit activity by interacting with brevilaterin. Furthermore, the SPR experiment confirmed this speculation in Figure 5d. As a different concentration of soybean phospholipid flowed over the fixed brevilaterin, the SPR response value reached a peak at 100 s and then decreased rapidly. The affinity constant was *K_D_* = 4.70 × 10^−4^ M, which proved brevilaterin can interact with soybean phospholipid through a rapid binding and rapid dissociation process.

Appropriate hydrophobic interaction is quite essential for the activity of an antibacterial peptide [61]. It was estimated that most antibacterial peptides have about 50% of hydrophobic amino acids residues on average, providing a structure foundation for the membrane action mechanism [62], but every coin has two sides. Therefore, the interaction effect in a practical application also caught our attention. A hydrophobic environment, especially liposoluble constituents in foods, may result in a loss of antibacterial activity of brevilaterin, or in an increase of the efficient dosage to food-borne bacteria or spoilage bacteria. In this research, brevilaterin have been proved to increase the loss in antibacterial activity caused by the interaction with soybean phospholipid, as previously reported, whilst also providing some evidence. Brevilaterins exhibited antibacterial activity against *S. aureus* in raw milk at 300 μg/mL [17], while *S. aureus* in skim milk can be significantly inhibited by 16 μg/mL of brevibacillin [9], and nisin showed a complete loss of antibacterial activity in foods, where it contained more than 1% of lectins [63].

## 4. Conclusions

In summary, this systematic work has contributed rich antibacterial data of brevilaterins and revealed the regularity beneath these data. We proved that brevilaterins are broad-spectrum antibacterial agents with good inhibitory and bactericidal activity. They are especially efficient against Gram-positive bacteria, comparable to commercial vancomycin and daptomycin. Spoilage bacteria from grain products, vegetables and fruits, soybean products, and animal foods would be generally inhibited by brevilaterins. Moreover, brevilaterins showed low application risk because they not only demonstrated good antibacterial activity on MRSA and VRE, as well as 70 strains of antibiotic-resistant isolated bacteria, but they also proved it is difficult to develop resistance with a small specific value of MBC/MIC (1~8). Although brevilaterin B and brevilaterin C have no significant activity difference due to the difference of one amino acid residue, brevilaterins can interact with negatively charged SDS and amphipathic soybean phospholipid for its positive charge and hydrophobicity structure, suggesting they are not suitable for use in a liposoluble food system. This solid theoretical foundation can be used as an activity handbook to provide accurate instructions for the food application of brevilaterins.

## Figures and Tables

**Table 1:** Quality control of MIC determination system.

QC Strains	MIC (µg/mL)						
	Test Results					QC Range	
	BB	BC	PB	Da	Van	PB	Da
*S. aureus* subsp. *aureus* ATCC 29213	1	1	-	1	1	-	0.12~1
*E. faecalis* ATCC 29212	1	1	-	4	1	-	1~4
*P. aeruginosa* ATCC 27853	32	32	2	-	-	0.25~2	-
*E. coli* ATCC 25922	16	16	2	-	-	0.5~4	-

BB, brevilaterin B; BC, brevilaterin C; PB, polymyxin B; Da, daptomycin; Van, vancomycin. QC range was referred to CLSI-M07 A9 [26].

**Table 2:** MIC and MBC values of brevilaterins against 50 strains of standard bacteria.

Genera	Species	MIC (μg/mL)					
		Brevilaterin B			Brevilaterin C		
		MIC	MBC	MBC/MIC	MIC	MBC	MBC/MIC
Gram-positive bacteria							
*Bacillus*	*Bacillus coagulans* (CICC 20138)	0.5	1	2	0.5	1	2
	*Bacillus megaterium* (CICC 10448)	0.5	0.5	1	0.5	0.5	1
	*Bacillus megaterium*	1	1	1	1	1	1
	*Bacillus cereus* (ATCC 11778)	1	2	2	1	1	1
	*Bacillus pumilus* (CICC 10900)	1	8	8	1	8	8
	*Bacillus fusiformis* (CICC 20463)	2	4	2	2	4	2
	*Bacillus subtilis* (CICC 10275)	2	4	2	2	4	2
	*Bacillus subtilis* subsp. *Subtilis* (ATCC 6051)	2	2	1	1	1	1
*Staphylococcus*	*Staphylococcus cohnii* subsp. *cohnii* (CICC 20742)	1	2	2	1	2	2
	*Staphylococcus aureus* (CICC 10001)	2	4	2	2	4	2
	*Staphylococcus epidermidis* (CICC 10436)	2	8	4	1	8	8
	*Staphylococcus aureus* (ATCC 25923)	1	4	4	1	2	2
	*Staphylococcus aureus* (ATCC 29213)	1	4	4	1	2	2
	*Staphylococcus aureus* subsp. *aureus* (ATCC 6538)	2	2	1	2	2	1
	*Methicillin-resistant and oxacillin-resistant Staphylococcus aureus* (ATCC 43300)	4	8	2	4	8	2
*Listeria*	*Listeria monocytogenes* (10403s)	1	4	4	1	8	8
	*Listeria monocytogenes* (ATCC 19115)	1	8	8	1	8	8
*Enterococcus*	*Vancomycin resistant enterococcus* (ATCC 51299)	1	2	2	1	2	2
	*Enterococcus faecalis* (ATCC 29212)	1	8	8	1	8	8
	*Enterococcus faecalis* (CICC 10396)	2	8	4	1	8	8
*Paenibacillus*	*Paenibacillus polymyxa* (CICC 20128)	16	32	2	16	32	2
*Micrococcus*	*Micrococcus luteus* (CICC 10269)	0.5	1	2	0.5	1	2
*Streptococcus*	*Streptococcus gallolyticus* (CICC 10203)	1	4	4	1	4	4
*Lactococcus*	*Lactococcus lactis* (CICC 20711)	1	2	2	1	2	2
*Leuconosto*	*Leuconostoc mesenteroides* (CICC 20074)	2	8	4	2	8	4
*Lactobacillus*	*Lactobacillus buchneri* (CICC 20015)	1	2	2	1	2	2
Gram-negative bacteria							
*Acinetobacter*	*Acinetobacter baumannii* (CICC 10980)	4	16	4	4	16	4
*Alcaligenes*	*Alcaligenes faecalis* (CICC 10981)	4	32	8	8	16	2
*Shewanella*	*Shewanella putrefaciens* (CICC 22940)	4	8	2	4	8	2
*Pseudomonas*	*Pseudomonas maltophilia* (CICC 20702)	16	32	2	8	16	2
	*Pseudomonas aeruginosa* (ATCC 9027)	32	128	4	16	128	8
	*Pseudomonas aeruginosa* (ATCC 27853)	32	128	4	32	128	4
	*Pseudomonas fluorescens* (ATCC 13525)	128	256	2	128	256	2
*Shigella*	*Shigella dysenteriae* (CICC 23829)	8	32	4	8	16	2
	*Shigella flexneri* (CICC 10865)	16	64	4	16	64	4
	*Shigella sonnei* (CICC 21535)	16	32	2	16	64	4
*Escherichia*	*Escherichia coli* (ATCC 25922)	8	16	2	8	32	4
	*Escherichia coli* (CMCC 44752)	16	32	2	16	32	2
*Klebsiella*	*Klebsiella pneumoniae* (CICC 10870)	16	32	2	16	32	2
*Cronobacter*	*Cronobacter sakazakii* (CICC 21560)	16	128	8	16	128	8
*Vibrio*	*Vibrio parahaemolyticus* (CICC 21528)	16	32	2	16	32	2
	*Vibrio cholerae* (CICC 23794)	16	32	2	16	32	2
*Citrobacter*	*Citrobacter freundii* (CICC 10404)	32	128	4	32	128	4
*Yersinia*	*Yersinia enterocolitica* (CICC 21565)	32	256	8	32	64	2
*Salmonella*	*Salmonella typhimurium* (CICC 21484)	32	128	4	16	32	2
	*Salmonella enterica* subsp. *enterica serovar typhimurium* (ATCC 14028)	32	64	2	32	64	2
*Enterobacter*	*Enterobacter aerogenes* (CICC 10293)	64	128	2	64	128	2
*Serratia*	*Serratia marcescens* (CICC 10898)	NI	NI	-	NI	NI	-
*Proteus*	*Proteus mirabilis* (CICC 21516)	NI	NI	-	NI	NI	-
	*Proteus vulgaris* (CICC 10866)	NI	NI	-	NI	NI	-

**NI**, value undetected even at 256 μg/mL.

**Table 3:** Summary of MIC and MBC value of brevilaterin B and C against standard bacteria.

Brevilaterin	Standard Indicators	MIC_50_	MIC_90_	MIC_R_	MBC_50_	MBC_90_	MBC_R_
		μg/mL					
BB	Gram-positive (*n* = 26)	1	2	0.5~16	2	8	0.5~128
	Gram-negative (*n* = 24)	16	>128	4~128	32	>128	8~256
	Total (*n* = 50)	4	32	0.5~128	8	128	0.5~256
BC	Gram-positive (*n* = 26)	1	2	0.5~16	4	8	0.5~32
	Gram-negative (*n* = 24)	16	>128	4~128	64	>128	8~128
	Total (*n* = 50)	4	32	0.5~128	8	128	0.5~256

MIC_50_, MBC_50_: MIC or MBC value to inhibit or kill 50% of test strains; MIC_90_, MBC_90_: MIC or MBC value to inhibit or kill 90% of test strains; MIC_R_, MBC_R_: MIC or MBC range to inhibit or kill all test strains.

**Table 4:** Summary of MIC and MBC values of brevilaterin B and C to isolated spoilage bacteria from different foods.

Brevilaterin	Isolation Source	MIC_50_	MIC_90_	MIC_R_	MBC_50_	MBC_90_	MBC_R_
		μg/mL					
BB	Vegetables and fruits (*n* = 14)	16	64	0.5~64	32	128	1~128
	Animal products (*n* = 19)	32	64	0.5~>256	64	256	2~>256
	Soybean products (*n* = 21)	16	32	0.5~64	32	128	0.5~128
	Grain products(*n* = 16)	1	64	0.5~64	4	128	1~256
	Total (*n* = 70)	16	64	0.5~>256	32	128	0.5~>256
BC	Vegetables and fruits (*n* = 14)	16	32	0.5~64	32	256	1~256
	Animal products (*n* = 19)	16	64	0.5~>256	64	256	2~>256
	Soybean products (*n* = 21)	16	64	0.5~64	32	128	0.5~128
	Grain products (*n* = 16)	1	64	0.5~64	4	128	1~256
	Total (*n* = 70)	16	64	0.5~>256	32	128	0.5~>256

MIC_50_, MBC_50_: MIC or MBC value to inhibit or kill 50% of test strains; MIC_90_, MBC_90_: MIC or MBC value to inhibit or kill 90% of test strains; MIC_R_, MBC_R_: MIC or MBC range to inhibit or kill all test strains.

## Data Availability

Data is contained within the article or Supplementary Material.

## Supplementary Materials

The following supporting information can be downloaded at: https://www.mdpi.com/article/10.3390/foods11192991/s1, Table S1: Culture medium and temperature of CICC standard bacteria; Table S2: MICs, MBCs and their ratios of brevilaterins against food spoilage bacteria from different foods; Table S3: MICs, MBCs and their ratios of brevilaterins to different types of resistant bacteria.
