# Supplementary material for: Antibacterial Regularity Mining Beneath the Systematic Activity Database of Lipopeptides Brevilaterins: An Instructive Activity Handbook for Its Food Application
Source: Foods. 2022 Sep 26;11(19):2991. doi: 10.3390/foods11192991 (PMC9563055; doi:10.3390/foods11192991)
Supplement: Supplementary file 1 [file foods-11-02991-s001.zip › foods-1915575-supplementary.pdf]

Table S1. Culture medium and temperature of CICC standard bacteria

Genera	Species	Medium	Temperature (°C)
Gram-positive bacteria			
Bacillus	*Bacillus coagulans* (CICC 20138)	CM0002	37
	Bacillus megaterium (CICC 10448)	CM0002	37
	Bacillus megaterium (B020)	CM0002	37
	Bacillus cereus (ATCC 11778)	CM0002	30
	Bacillus pumilus (CICC 10900)	CM0002	37
	Bacillus fusiformis (CICC 20463)	CM0033	37
	Bacillus subtilis (CICC 10275)	CM0002	37
	Bacillus subtilis subsp. *Subtilis* (ATCC 6051)	CM0002	30
Staphylococcus	*Staphylococcus cohnii* subsp. *cohnii* (CICC 20742)	CM0002	37
	Staphylococcus aureus (CICC 10001)	CM0350	37
	Staphylococcus epidermidis (CICC 10436)	CM0002	37
	Staphylococcus aureus (ATCC 25923)	CM0002	37
	Staphylococcus aureus (ATCC 29213)	CM0847	37
	Staphylococcus aureus subsp. *aureus* (ATCC 6538)	CM0002	37
	Methicillin-resistant and oxacillin-resistant Staphylococcus aureus (ATCC 43300)	CM0847	37
Listeria	*Listeria monocytogenes* (10403s)	CM0217	37
	Listeria monocytogenes (ATCC 19115)	CM0217	37
Enterococcus	*Vancomycin resistant enterococcus* (ATCC 51299)	CM0217 +4 µg/mL Vaconomycin	37
	Enterococcus faecalis (ATCC 29212)	CM0847	37
	Enterococcus faecalis (CICC 10396)	CM0847	37
Paenibacillus	*Paenibacillus polymyxa* (CICC 20128)	CM0002	28
Micrococcus	*Micrococcus luteus* (CICC 10269)	CM0002	30
Streptococcus	*Streptococcus gallolyticus* (CICC 10203)	CM0195	30
Lactococcus	*Lactococcus lactis* (CICC 20711)	CM0006	30
Leuconosto	*Leuconostoc mesenteroides* (CICC 20074)	CM0043	28
Lactobacillus	*Lactobacillus buchneri* (CICC 20015)	CM0005	30
Gram-negative bacteria			
Acinetobacter	*Acinetobacter baumannii* (CICC 10980)	CM0002	37
Alcaligenes	*Alcaligenes faecalis* (CICC 10981)	CM0002	37

Shewanella	*Shewanella putrefaciens* (CICC 22940)	CM0847	30
Pseudomonas	*Pseudomonas maltophilia* (CICC 20702)	CM0002	30
	Pseudomonas aeruginosa (ATCC 9027)	CM0002	37
	Pseudomonas aeruginosa (ATCC 27853)	CM0847	37
	Pseudomonas fluorescens (ATCC 13525)	CM0002	30
Shigella	*Shigella dysenteriae* (CICC 23829)	CM0051	37
	Shigella flexneri (CICC 10865)	CM0002	36
	Shigella sonnei (CICC 21535)	CM0002	36
Escherichia	*Escherichia coli* (ATCC 25922)	CM0002	37
	Escherichia coli (CMCC 44752)	CM0002	37
Klebsiella	*Klebsiella pneumoniae* (CICC 10870)	CM0002	30
Cronobacter	*Cronobacter sakazakii* (CICC 21560)	CM0002	36
Vibrio	*Vibrio parahaemolyticus* (CICC 21528)	CM0111	36
	Vibrio cholerae (CICC 23794)	CM0111	37
Citrobacter	*Citrobacter freundii* (CICC 10404)	CM0002	36
Yersinia	*Yersinia enterocolitica* (CICC 21565)	CM0002	36
Salmonella	*Salmonella typhimurium* (CICC 21484)	CM0002	36
	Salmonella enterica subsp. *enterica serovar typhimurium* (ATCC 14028)	CM0002	37
Enterobacter	*Enterobacter aerogenes* (CICC 10293)	CM0002	30
Serratia	*Serratia marcescens* (CICC 10898)	CM0002	30
Proteus	*Proteus mirabilis* (CICC 21516)	CM0002	36
	Proteus vulgaris (CICC 10866)	CM0002	30

Medium information referred to http://www.china-cicc.org/

Table S2. MICs, MBCs, and their ratios of brevilaterins against food spoilage bacteria from different foods

Foods category	Food source	Bacterial number	Gram species	BB			BC		
				MIC	MKC	MBC/MIC	MIC	MKC	MBC/MIC
Animal foods	milk	U-2-2	G^+	0.5	2	4	1	4	4
	yogurt	Y1-4-3	G^+	0.5	2	4	0.5	2	4
	ham	Q7-Y-1	G^+	1	4	4	0.5	4	8
	saury	F2-3-5	G^+	1	4	4	1	2	2
	yogurt	Y1-4-2	G^+	1	2	2	0.5	4	8
	saury	F2-3-2	G^-	16	16	1	16	16	1
	milk	U1-4-4	G^-	16	128	8	16	128	8
	quail egg	E4-4-3	G^-	32	32	1	16	16	1
	beef	Q2-3-3	G^-	32	32	1	32	32	1
	beef	Q2-4-3	G^-	32	128	4	32	128	4
	chicken	Q3-4-4	G^-	32	256	8	32	128	4
	chicken	Q3-4-6	G^-	32	32	1	16	32	2
	ham	Q7-3-2	G^-	32	64	2	16	64	4
	yogurt	Y1-4-1	G^-	32	64	2	32	64	2
	pork	Q1-3-1	G^-	64	64	1	64	64	1
	milk	U-2-1	G^-	64	64	1	32	128	4
	milk	U-2-3	G^-	64	128	2	64	128	2
	milk	U1-4-3	G^-	64	256	4	64	256	4
	beef	Q2-3-1	G^-	NI	NI	-	NI	NI	-
Soybean products	Marinated Tofu	D1-0-1-1	G^+	0.5	2	4	0.5	1	2
	Smoked bean curd	D3-0-1-1	G^+	0.5	0.5	1	0.5	0.5	1
	fermented bean curd	I2-3-2	G^+	0.5	2	4	0.5	1	2
	Smoked bean curd	D3-0-1-2	G^+	1	1	1	0.5	1	2
	dried bean curd	D4-0.5-2-1	G^+	1	4	4	1	4	4
	dried bean curd	D4-0.5-2-3	G^+	1	4	4	0.5	4	8
	Smoked bean curd	D3-0.5-2-1	G^+	4	8	2	4	8	2
	Lactone tofu	G1-3-1	G^-	16	16	1	16	16	1
	Lactone tofu	D2-0-Y-1	G^-	16	128	8	16	128	8
	Lactone	D2-0-Y-2	G^-	16	32	2	16	32	2

	tofu								
	Lactone tofu	D2-0.5-1-2	G^-	16	64	4	16	32	2
	soybean milk	D5-3-3	G^-	16	32	2	16	32	2
	fermented bean curd	I2-3-3	G^-	16	64	4	16	32	2
	Marinated Tofu	D1-0-1-2	G^-	32	128	4	32	64	2
	soybean milk	D5-3-2	G^-	32	64	2	32	64	2
	fermented bean curd	I2-3-1	G^-	32	32	1	16	16	1
	fermented bean curd	I2-3-4	G^-	32	32	1	32	32	1
	fermented bean curd	I2-4-1	G^-	32	32	1	16	16	1
	Lactone tofu	D1-0.5-1-1	G^-	64	128	2	64	128	2
	Lactone tofu	D2-0.5-1-1	G^-	64	128	2	64	64	1
	dried bean curd	D4-0.5-2-2	G^-	64	128	2	64	128	2
Grain foods	Glutinous rice products	N-Y-1	G^+	0.5	2	4	0.5	1	2
	Glutinous rice products	N-Y-2	G^+	0.5	2	4	0.5	2	4
	noodles	Tf-1	G^+	0.5	1	2	0.5	2	4
	bread	Q5-3-1	G^+	0.5	1	2	1	2	2
	purple potatoes	Z-2-1	G^+	0.5	2	4	0.5	1	2
	noodles	Q6-3-3	G^+	1	2	2	0.5	2	4
	steamed bun	Q4-4-1	G^+	1	4	4	1	4	4
	steamed bun	Q4-4-3	G^+	1	2	2	1	2	2
	purple potatoes	Z-2-2	G^+	1	4	4	1	4	4
	noodles	Q6-3-2	G^-	16	16	1	16	32	2
	fresh noodles	O-Y-2	G^-	32	64	2	32	32	1

	fresh noodles	O-1	G^-	32	128	4	16	64	4
	fresh noodles	TY-1	G^-	32	128	4	16	128	8
	Kimbob	B-3-3	G^-	32	128	4	32	128	4
	steamed bun	Q4-4-2	G^-	64	256	4	64	256	4
	Kimbob	B-3-1	G^-	64	128	2	64	128	2
Vegetables and fruits	mango	M-Y-1	G^+	0.5	4	8	0.5	2	4
	cabbage	C2-4-5	G^+	0.5	4	8	0.5	2	4
	mango	M-1-1	G^+	1	1	1	0.5	1	2
	lettuce	C-2-3	G^+	1	4	4	1	4	4
	pickle	K-1-2	G^+	1	2	2	1	2	2
	pitaya	H-Y-2	G^-	16	32	2	16	32	2
	mango	M-Y-2	G^-	16	16	1	16	32	2
	apple	P-1-1	G^-	16	64	4	16	64	4
	cabbage	C2-4-4	G^-	16	32	2	16	16	1
	lettuce	C-3-2	G^-	32	128	4	32	128	4
	pickle	K-2-1	G^-	32	128	4	32	256	8
	pitaya	H-Y-1	G^-	64	128	2	32	64	2
	apple	P-1-2	G^-	64	64	1	32	64	2
	Lettuce	C-2-1	G^-	64	128	2	64	256	4

Table S3. MICs, MBCs, and their ratios of brevilaterins to different types of resistant bacteria

Antibiotic resistance classification	Strains number	Gram species	BB			BC		
			MIC	MKC	MBC/MIC	MIC	MKC	MBC/MIC
Methicillin	22-02-2	G^+	0.5	1	2	0.5	0.5	1
	22-02-4	G^+	0.5	2	4	0.5	2	4
	22-03-4	G^+	0.5	2	4	0.5	1	2
	22-05-3	G^+	0.5	2	4	0.5	2	4
	22-03-1	G^+	1	2	2	1	4	4
	03-02-2	G^+	4	8	2	4	8	2
	03-04-1	G^+	4	4	1	4	4	1
	03-05-1	G^+	4	16	4	4	8	2
	22-03-2	G^+	4	8	2	4	16	4
	22-04-2	G^+	4	16	4	4	16	4
	03-02-3	G^-	8	16	2	4	16	4
	22-03-3	G^-	8	32	4	8	32	4
	03-03-1	G^-	16	128	8	16	64	4
	22-04-1	G^-	16	16	1	8	16	2
	22-04-3	G^-	16	128	8	16	16	1
	22-04-4	G^-	16	32	2	16	32	2
	22-05-1	G^-	16	64	4	16	64	4
	22-05-2	G^-	16	32	2	16	32	2
	03-02-1	G^-	32	128	4	16	128	8
	04-02-1	G^+	0.5	1	2	0.5	1	2
	04-01-1	G^+	1	4	4	0.5	4	8
	04-02-3	G^+	1	1	1	0.5	4	8
	04-01-5	G^+	2	4	2	2	8	4
	04-02-4	G^+	2	2	1	1	1	1
Penicillin	04-06-1	G^+	2	4	2	2	8	4
	04-02-5	G^+	4	16	4	2	8	4
	04-01-4	G^-	8	64	8	8	64	8
	04-02-2	G^-	16	16	1	8	32	4
	04-01-2	G^-	32	128	4	32	64	2
	04-01-3	G^-	32	256	8	16	128	8
	01-03-1	G^+	0.5	4	8	0.5	4	8
	01-12-1	G^+	0.5	4	8	0.5	4	8
	02-10-2	G^+	1	2	2	1	2	2
	02-11-1	G^+	1	4	4	0.5	4	8
vancomycin	01-02-1	G^-	4	8	2	4	16	4
	01-04-5	G^+	4	8	2	2	4	2
	01-11-1	G^+	4	4	1	2	4	2
	02-02-5	G^+	4	4	1	4	4	1
	01-04-1	G^-	8	16	2	8	16	2

	01-04-3	G^-	16	32	2	16	64	4
	01-06-1	G^-	64	128	2	64	64	1
Teicoplanin	27-02-2	G^+	2	8	4	2	8	4
	27-05-1	G^+	4	8	2	4	4	1
	27-05-2	G^+	4	4	1	1	4	4
	27-02-3	G^+	16	64	4	16	64	4
Ciprofloxacin	06-04-1	G^+	0.5	1	2	0.5	2	4
	26-02-1	G^+	2	4	2	2	4	2
	06-02-1	G^-	16	32	2	16	64	4
	06-04-2	G^-	16	64	4	16	128	8
Meropenem	25-02-1	G^+	2	16	8	2	16	8
	25-03-1	G^+	2	16	8	2	16	8
	25-02-2	G^+	4	4	1	2	4	2
	05-02-1	G^-	8	8	1	16	16	1
Rifampicin	24-02-3	G^+	0.5	4	8	0.5	4	8
	24-04-3	G^+	1	1	1	0.5	0.5	1
Ciprofloxacin+penicillin	06-06-1	G^+	2	8	4	2	4	2
Meropenem+methicillin	05-03-1	G^+	2	4	2	2	4	2
	05-01-1	G^+	4	8	2	4	8	2
	03-06-1	G^-	16	32	2	16	16	1
Meropenem+Vancomycin	02-02-2	G^+	2	4	2	2	8	4
	02-03-2	G^+	2	8	4	2	8	4
	02-01-1	G^-	4	32	8	4	32	8
	02-02-1	G^-	4	32	8	4	32	8
Teicoplanin+Meropenem	27-02-1	G^-	8	32	4	8	16	2
	27-02-4	G^-	16	32	2	16	32	2
Vancomycin+Rifampicin	21-03-1	G^+	0.5	1	2	0.5	1	2
	24-04-2	G^+	4	4	1	2	2	1
	24-02-1	G^-	8	64	8	8	64	8
	24-02-2	G^-	8	64	8	8	64	8
	24-04-1	G^-	32	32	1	32	32	1
